# Cartas al editor

**DOI:** 10.7705/biomedica.8620

**Published:** 2026-08-20

Cali, 5 de agosto del 2026

Señores

Revista *Biomédica*

Instituto Nacional de Salud 

Bogotá, D. C., Colombia

Estimados señores:

Adjunto carta al editor titulada «Enfermedades huérfanas en Colombia: la imprescindible perspectiva quirúrgica pediátrica» con la autoría mía y de Manuela Eusse Soto.

Reciban un cordial saludo.

Atentamente,

Luis Mauricio Figueroa-Gutiérrez^1,2^https://orcid.org/0000-0002-7959-1768

^¹^ Sección de Cirugía Pediátrica, Escuela de Medicina, Universidad del Valle, Cali, Colombia.

^²^ Servicio de Cirugía Pediátrica, Clínica Comfamiliar, Pereira, Colombia. figueroa.luis@correounivalle.edu.co

Manuela Eusse Soto https://orcid.org/0000-0002-7527-364X

Universidad Libre, Cali, Colombia manuelaeusses@gmail.com

## Enfermedades huérfanas en Colombia: la imprescindible perspectiva quirúrgica pediátrica

Hemos leído con interés el editorial «Enfermedades huérfanas o raras en Colombia: situación actual y sugerencias para un futuro mejor» ^1^, en el que el autor expone de manera muy clara las dificultades relacionadas con este grupo de enfermedades en nuestro país, empezando por la confusión en la nomenclatura utilizada y en su definición, la cual debería considerar enfermedades con una frecuencia de uno por cada 2000 nacidos vivos, lo que incrementaría la cobertura del sistema de salud para un mayor número de pacientes.

Debe tenerse en cuenta que, además del enfoque presentado por el autor, en el cual predominan entidades de manejo médico o clínico-genéticas, metabólicas o que requieren tratamientos farmacológicos de alto costo, como la terapia génica o de reemplazo enzimático-, existe un grupo de enfermedades, algunas incluidas en el listado y otras que aún no han sido tenidas en cuenta y cuya primera línea de tratamiento no es un fármaco, sino una corrección quirúrgica neonatal o pediátrica oportuna ^2^.

Entre las enfermedades incluidas, se encuentran la enfermedad de Hirschsprung (Q43.1) y las malformaciones anorrectales con fístula (Q42.2) y sin fístula (Q42.3). Sin embargo, la atresia esofágica sin fístula (Q39.0) y con fístula (Q39.1), aunque son estadísticamente igual de frecuentes que las anteriores, solamente que aparecen asociadas con síndromes en el código Q87.8. Asimismo, anomalías congénitas de gran complejidad, como la persistencia de la cloaca, las valvas de uretra posterior y el complejo extrofia vesical-epispadias, aún no han sido incluidas, a pesar de ser condiciones con frecuencias aproximadas de uno por cada 30 000 a 50 000 nacidos vivos ^2^.

Por otra parte, el complejo onfalocele, ano imperforado, extrofia de cloaca y anomalías espinales *(omphalocele, exstrophy of the cloaca, imperforate anus, spinal defects,* GEIS) y otras condiciones, como el síndrome de Ochoa, sí aparecen en el listado. Esto confirma lo planteado por el autor respecto a la necesidad de que, durante la actualización del listado que se realiza cada dos años, sean incorporadas estas enfermedades, consideradas extremadamente raras en algunos países ^1^^,^^2^.

Además, debe tenerse en cuenta que, debido a su gran complejidad, la intervención quirúrgica inicial y los procedimientos posteriores que puedan requerirse no siempre logran la corrección completa del problema. Por esta razón, estos pacientes necesitan seguimiento en centros especializados por grupos interdisciplinarios durante toda su vida, incluida la transición hacia la atención en la edad adulta ^3^.

En cuanto a las barreras para el diagnóstico temprano y el inicio oportuno del tratamiento quirúrgico, el desconocimiento de estas enfermedades por parte del personal de salud retrasa en muchas ocasiones su identificación. Además, los objetivos de la Ley 1392 de 2010, relacionados con el desarrollo de guías de atención y centros de excelencia, y de la Ley Estatutaria 1751 de 2015, que establece la atención prioritaria y especial de estos pacientes, no se han alcanzado plenamente. Esta situación condena a los pacientes y a sus familias a enfrentar múltiples restricciones administrativas y económicas que impiden que sean tratados específicamente por cirujanos pediatras y urólogos pediatras con el entrenamiento requerido para el manejo de estas condiciones especiales en centros de referencia ^4^.

Con respecto a la disponibilidad de recursos físicos y humanos competentes, además de las estrategias existentes en los Estados Unidos y en Europa, en Latinoamérica se conformó desde 2019, el Grupo Brasileño Colaborativo Interinstitucional para la corrección del complejo extrofia vesical-epispadias mediante la técnica de Kelly. De este grupo hacen parte cirujanos pediatras y urólogos pediatras de Colombia, quienes se han desplazado a más de veinte estados de Brasil y, en nuestro país, a las ciudades de Cali y Barranquilla, para intervenir a pacientes con esta anomalía, considerada una de las más complejas del sistema genitourinario ^5^.

Esta estrategia ha permitido que más de 150 pacientes se hayan beneficiado de la atención por parte de un grupo de excelencia, con mejores resultados derivados de la corrección mediante una técnica quirúrgica estandarizada y validada ^5^.

Por último, la creación de redes no solamente debe enfocarse en la atención quirúrgica especializada -por ejemplo, mediante centros de excelencia para la atención de anomalías genitourinarias y colorrectales complejas-, sino también, en la conformación de grupos de apoyo entre pares. Estos espacios permitirían que los padres y cuidadores compartieran sus experiencias y vivencias y que, como comunidad, pudieran gestionar reuniones nacionales de pacientes con estas anomalías. Todo lo anterior redundaría en una mejor atención para los próximos niños que presenten estas enfermedades ^6^.

En conclusión, son varias las tareas que deben desarrollarse con respecto a las enfermedades huérfanas en nuestro país. Desde la academia, los diferentes programas universitarios responsables de la formación de profesionales de la salud deben comprometerse a incluir estas enfermedades dentro de sus currículos, con el propósito de garantizar su diagnóstico y la derivación oportuna hacia centros de excelencia.

Para las condiciones cuyo tratamiento implica una gran cantidad de recursos económicos, tecnológicos y científicos, deben desarrollarse políticas públicas que permitan considerar estos casos como lo define la literatura: «pacientes con necesidades especiales de atención en salud», y garantizar que se actúe con prioridad y determinación ^7^.

La conformación de centros de referencia organizados por sistemas -vía aérea y esófago, hepatobiliar y gastrointestinal, genitourinario y colorrectal, entre otros-, como existen en otros países, permitiría focalizar los casos. Se ha demostrado que esta concentración favorece mejores resultados, y una atención más segura y estandarizada ^8^.

Asimismo, la organización de los pacientes, sus padres y cuidadores en redes de apoyo, contribuiría a lograr una mayor difusión y visibilización de estas comunidades mediante las redes sociales y otros medios. Como sucede en otras regiones, esto permitiría realizar reuniones de asociaciones que impacten positivamente el tratamiento, mediante la propuesta de proyectos de ley y guías de manejo elaboradas desde la perspectiva de los pacientes y sus cuidadores ^6^^,^^9^.

Por último, el listado debe ser más amplio e incluyente, ya que quienes sufren las consecuencias de permanecer excluidos suelen ser pacientes con escasos recursos, secuelas graves a largo plazo y pocas oportunidades de alcanzar una mejor calidad de vida.
